# PET/CT F18-FDG with soft tissue plasmacytomas in multiple myeloma

**DOI:** 10.1186/s41824-021-00100-7

**Published:** 2021-03-31

**Authors:** Alejandro Martí, Sarai Morón, Sandra Chinchilla, Eliana González

**Affiliations:** 1 Department of Nuclear Medicine and PET/CT, National Cancer Institute and PET/CT Idime, Bogotá, Colombia; 2 Nuclear Medicine, Valledupar, Colombia; 3 Department of Pathology, National Cancer Institute, Bogotá, Colombia; 4 Nuclear Medicine, Sanitas Foundation, Bogotá, Colombia

**Keywords:** F18-FDG PET/CT, Multiple myeloma, Soft tissue, Plasmacytoma

## Abstract

Multiple myeloma is characterized by malignant proliferation of clonal plasma cells. Usually, appears as a generalized disease but it can present as solitary bone plasmacytoma or a solitary soft tissue mass or extramedullary plasmacytoma. In the case of extramedullary involvement, it could present as soft tissue plasmacytomas and the prognosis is poor. The 18F-FDG PET/CT could be a valuable tool for characterization of the medullary and extramedullary involvement. We present a case of F18-FDG PET/CT with extramedullary involvement with soft tissue plasmacytomas in the setting of MM.

## Case presentation

**Fig. 1:**
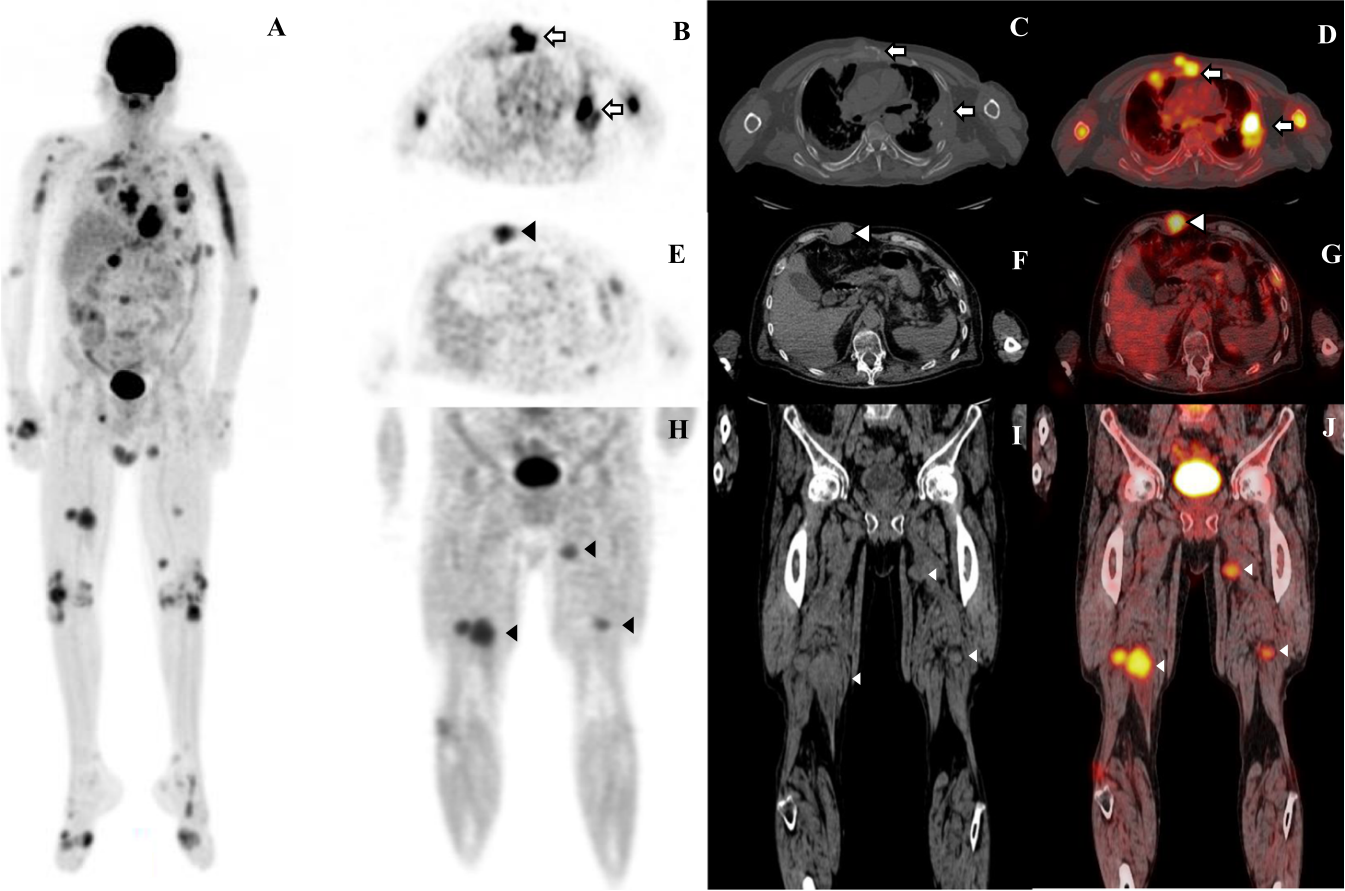
**a** MIP image shows multiple soft tissue masses with high FDG uptake and hypermetabolic lytic bone lesions. **b**-**d** On axial images, lytic lesions in sternum and ribs with soft-tissue mass and increased FDG uptake (white arrows). **e**-**g** Hypermetabolic subcutaneous mass in right abdomen wall with FDG uptake (arrowheads). **h**-**j** In extremities, subcutaneous lesions with high uptake of FDG were seen (arrowheads)

We present a case of a patient, a 54-year-old man that was diagnosed with multiple myeloma. He referred mass sensation with progressive growth located in thorax, abdomen, and upper and lower extremities. A whole body 18F-FDG PET/CT (positron emission tomography/computed tomography) was performed as part of initial staging. This showed multiple soft tissue masses in extremities, abdomen, and thorax wall with high FDG uptake and hypermetabolic lytic bone lesions (Fig. 1a). On axial images, increased FDG uptake noted in lytic lesions in sternum and ribs with soft-tissue mass and SUV_max_ of 9.5 and 5, respectively (Fig. 1b-d). In addition, multiple hypermetabolic subcutaneous masses located in thorax and abdomen wall and extremities were shown. In the right abdomen wall, a subcutaneous lesion with FDG uptake and SUV_max_ of 5 were demonstrated (Fig. 1e-g). In extremities with more involvement in the lower, subcutaneous lesions with high uptake of FDG were seen (Fig. 1h-j).

**Fig. 2:**
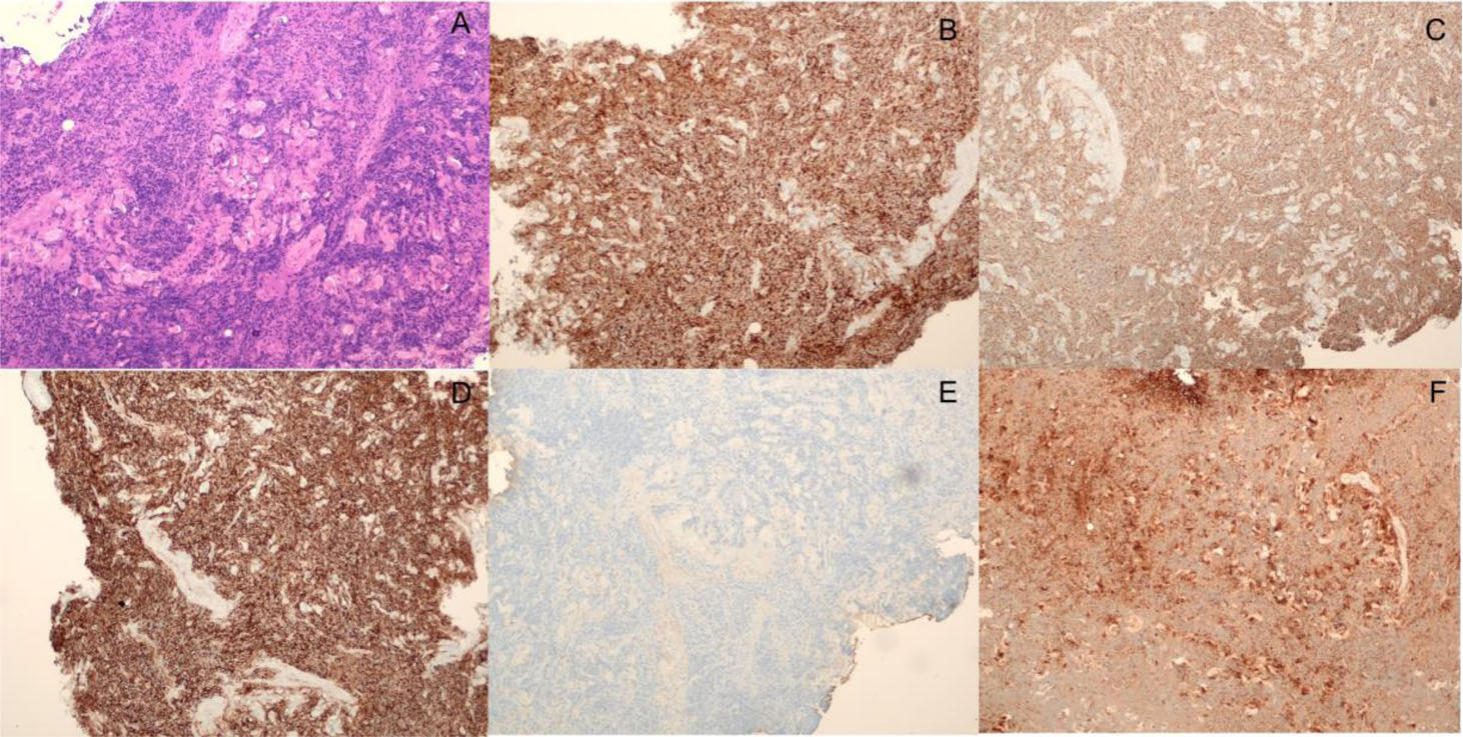
**a** Histology revealed monomorphic proliferation of atypical cells with basophilic nucleus. CD38 and CD138 immunoreactivity was found, respectively, confirming plasma cell differentiation (**b**-**c**), and CD56 aberrant expression is identified in plasma cells (**d**). Chain restriction without kappa light chain expression (**e**) and monotypic lambda chain expression (**f**)

A sonographic biopsy was performed in the abdominal soft tissue mass, and the results were monomorphic proliferation of atypical cells with basophilic nucleus (Fig. 2a). CD38 and CD138 immunoreactivity was found, respectively, confirming plasma cell differentiation (Fig. 2b-c) and CD56 aberrant expression is identified in plasma cells (Fig. 2d). Chain restriction without kappa light chains expression (Fig. 2e) and monotypic lambda chain expression (Fig. 2f). The histopathology confirmed soft tissue plasmacytomas.

## Discussion

Multiple myeloma (MM) is a neoplastic plasma-cell disorder that is characterized by clonal proliferation of malignant plasma cells in the bone marrow microenvironment (Palumbo and Anderson 2011). Extramedullary multiple myeloma (EMM) is a less frequent manifestation, where myeloma cells become independent of bone marrow microenvironment, infiltrate other organs, and patients could present involvement of lymph nodes, skin, soft tissues, central nervous system, thoracoabdominal organs, effusions, or any other anatomic sites (Bhutani et al. 2020). EMM can be present either at the time of initial diagnosis (primary EMM) or at the time of relapse (secondary EMM) (Usmani et al. 2012). The reported incidence of EMM ranges from 7 to 18% and the soft-tissues involvement in MM can have two different origins: direct extension from skeletal tumors when they disrupt the cortical bone or hematogenous metastatic spread (Bladé et al. 2012). This results from the extramedullary spread in MM and consists of single or multiple large highly vascularized subcutaneous nodules (Bladé et al. 2011). Moreover, patients with soft tissue related extramedullary release had significantly poorer overall survival (Pour et al. 2014).

The role of imaging in the work-up of patients with MM is aimed at allowing the recognition of both the effects of myeloma cells on the skeletal system and the presence of extramedullary disease (Nanni and Zamagni 2019). Over the last several decades, F18-FDG-PET/CT and magnetic resonance imaging (MRI) have shown incremental value in the management of patients with MM (Shah and Oldan 2017).

F18-FDG PET/CT can help to identify areas of metabolic activity in whole body that represent clonal plasma cell proliferation while MRI is particularly well suited for the imaging of bone marrow (Ferraro et al. 2015). Few cases are reported about soft tissue involvement in multiple myeloma in F18-FDG PET/CT (Ak and Gülbas 2007; Lapa et al. 2014) and this could be considered as a valuable diagnosis tool in particular for the detection of paramedullary and extramedullary soft tissue masses or solid organ involvement (Cavo et al. 2017).

## Conclusion

This case represents an unusual presentation of multiple myeloma in 18F-FDG PET/CT and emphasizes on the value of whole-body images for characterization of the medullary and extramedullary involvement.

## Data Availability

Not applicable
